# Trends in hypertension prevalence, awareness, treatment, and control in the Thai population, 2004 to 2020

**DOI:** 10.1186/s12889-024-20643-1

**Published:** 2024-11-13

**Authors:** Wichai Aekplakorn, Suwat Chariyalertsak, Pattapong Kessomboon, Sawitri Assanangkornchai, Surasak Taneepanichskul, Allison Goldstein, Danielle Cazabon, Nareemarn Neelapaichit, Obehioye Aimiosior

**Affiliations:** 1 Department of Community Medicine, Faculty of Medicine Ramathibodi Hospital, Mahidol University, Thailand University, Rama VI Rd., Ratchathewi, Bangkok, Thailand; 2 Faculty of Public Health, Chiang Mai University, Chiang Mai, Thailand; 3 Faculty of Medicine, Khon Kaen University, Khon Kaen, Thailand; 4 Epidemiology Unit, Faculty of Medicine, Prince of Songkla University, Songkhla, Thailand; 5 College of Public Health Sciences, Chulalongkorn University, Bangkok, Thailand; 6 Resolve To Save Lives, 85 Broad Street, Suite 1626, New York, NY USA; 7 Ramathibodi School of Nursing, Faculty of Medicine, Ramathibodi Hospital, Mahidol, Thailand

**Keywords:** Cross sectional study, Prevalence, Awareness, Control, Hypertension

## Abstract

**Background:**

Under Thailand’s universal health coverage every citizen has access to primary care including free hypertension treatment. This study describes temporal trends in hypertension prevalence, awareness, treatment, and control in Thailand.

**Methods:**

Data were analyzed from four survey cycles of Thailand’s National Health Examination Survey (NHES), between 2004 and 2019–2020. The NHES is a nationally-representative cross-sectional survey conducted every five years using a multistage probability sample. Hypertension was defined as systolic blood pressure > = 140 or diastolic blood pressure > = 90 mmHg or currently taking antihypertensive medicines; blood pressure control was defined as < 140/90 mmHg for patients without diabetes and < 130/80 mmHg for those with diabetes.

**Results:**

In 2019–2020, age-standardized hypertension prevalence in Thailand was 25.7% (24.6% females, 26.8% males). Among people with hypertension, 51.5% were aware of their diagnosis, 47.9% were treated, and 22.7% had controlled blood pressure. Age-standardized hypertension prevalence remained relatively unchanged in Thai adults from 2004 to 2019–2020, however trends varied by age group. Hypertension control increased from 8.8% in 2004 to a peak of 30% in 2014 but dropped to 22.7% by 2019–2020. Hypertension awareness increased from 30.7 to 55.8% between 2004 and 2014, but decreased to 51.5% by 2019–2020.

**Conclusion:**

Hypertension prevalence in Thailand has remained high over the past 15 years. Despite universal health coverage, hypertension awareness has not improved and blood pressure control has decreased in the past five years. An urgent and concerted public health response is needed to improve diagnosis and control of hypertension to prevent avoidable cardiovascular disease.

**Supplementary Information:**

The online version contains supplementary material available at 10.1186/s12889-024-20643-1.

## Background

Cardiovascular disease (CVD) is the leading cause of mortality in Thailand and is responsible for approximately one quarter of all deaths [1]. The annual prevalence of stroke cases increased from approximately 500 to 800 cases per 100,000 population between 2017 and 2023 and the age-standardized mortality rate of stroke was 63 per 100,000 person-years [2, 3]. Uncontrolled high blood pressure, or hypertension, is a major risk factor for CVD and is on the rise among the adult Thai population despite the country’s strong primary health care system. One in four Thai adults currently has hypertension and less than 20% of adults living with hypertension have it under control. Uncontrolled hypertension is implicated in more than 50,000 deaths annually in Thailand; two thirds of stroke cases and half of ischemic heart disease (IHD) cases are attributed to hypertension [4]. 

The purpose of the present study is to analyze data from the third, fourth, fifth and sixth cycles of Thailand’s National Health Examination Survey (NHES), from 2004 to 2019–2020, to determine trends in hypertension prevalence, awareness, treatment, and control in Thailand. A secondary objective was to analyze factors measured in the same surveys that are associated with these hypertension outcomes. Identifying factors associated with hypertension outcomes will allow the health care system to identify subgroups for targeted hypertension control interventions, and ultimately improve the management and control of hypertension.

## Methods

Thailand’s National Health Examination Survey (NHES) is a cross-sectional survey conducted every five years that collects data on the health of Thai population aged 15 years and older, using a multistage probability sample. For the current analysis, data from four survey cycles conducted from 2004 to 2019–2020 were analyzed. Random sampling is performed at each stage: during the first stage, five provinces are selected within each of four regions of Thailand; in the second stage, three to five districts are selected within the sampled provinces based on population size; and in the third stage, villages in rural areas and Enumeration units in urban areas are selected within the sampled districts. The fourth and final stage included the selection of individuals in the selected geographic areas to adequately represent five age groups (< 15, 15–59, 60–69, 70–79, >=80 years). For this analysis, only data from adults aged *≥* 20 years was included.

### Data collection

NHES data were collected through interviews, physical examination (blood pressure, and anthropometry), and obtaining blood samples for laboratory analysis [5]. Measurement of blood pressure, weight and height was performed by nurses trained for the field surveys. Subjects were interviewed in a community setting by trained nurses using standardized questionnaires to obtain information on demographics, education, health-related behaviors (smoking, drinking, and physical activity), area of residence, wealth index, and prior diagnosis of diabetes, hypertension, or CVD. Education level was categorized as less than primary, or secondary or greater and physical activity level was measured by using the WHO global physical activity questionnaire and classified as moderate to vigorous and low intensity. Wealth index is a composite measure for household socio-economic status. The index is derived from reporting ownership of selected assets, including: television, warm water, air conditioning, etc. The score was categorized into quintiles with 1 as poorest and 5 as richest. Body mass index (BMI) was calculated by dividing body weight (kg) by the square of height (meter).

### Participants

The analyses were restricted to adults aged 20 years or older who completed a study interview and examination (*n* = 100,230: 39,092 in 2004; 20,418 in 2009; 19,415 in 2014; and 21,305 in 2019–2020). Participants who were age < 20-year-old, a total of 5,168 (1217, 1109, 1349, and 1493 respectively) were excluded.

### Blood pressure measurement

For each survey cycle, field research assistants measured the blood pressure (BP) of participants using a validated automatic BP monitor, Omron model HEM 7117 (Omron Inc., Kyoto, Japan). The measurements were taken after the participants had rested for five minutes and while they were seated. BP was measured three times, with measurements separated by one minute according to the standard procedure [6]. The mean of the 2nd and 3rd systolic BP (SBP) and diastolic BP (DBP) measurements were calculated and used in the analysis to represent participants’ mean SBP and DBP, respectively.

Hypertension was defined as those participants having a mean SBP ≥ 140 mmHg or mean DBP ≥ 90 mmHg, or as self-report of having taken medication to lower their blood pressure in the prior two weeks [7]. 

### Outcomes

The primary outcomes were prevalence of hypertension among all adults and control of hypertension among all adults with hypertension and among treated. Hypertension prevalence was defined as the proportion of participants whose blood pressure measured SBP *≥* 140mmHg or DBP *≥* 90mmHg divided by the denominator of all participants. The primary outcome of proportion with controlled hypertension was defined for non-diabetic participants as the number of treated participants whose blood pressure measured SBP < 140mmHg and DBP < 90mmHg divided by the denominator of all non-diabetic participants with hypertension. For individuals with diabetes, proportion with controlled blood pressure was the number of treated participants with SBP < 130 mmHg and DBP < 80 mm Hg divided by the denominator of all diabetic participants with hypertension. Proportion with controlled hypertension among treated adults for non-diabetic participants was defined as the number of treated participants whose blood pressure measured SBP < 140mmHg and DBP < 90mmHg divided by the denominator of all non-diabetic treated participants. For individuals with diabetes, proportion with controlled blood pressure among treated adults was the number of treated participants with SBP < 130 mmHg and DBP < 80 mm Hg divided by the denominator of all treated diabetic participants [8]. 

Secondary outcomes were awareness of hypertension and treatment of hypertension. Awareness of hypertension was defined as an affirmative response by participants to the question “Have you ever been told by a doctor or other health worker that you have hypertension?”. Treatment of hypertension was determined by those who were aware they had hypertension and answered yes to the question “In the past two weeks, have you taken any drugs (medication) for raised blood pressure prescribed by a doctor or other health worker?”. The denominator of all persons with hypertension was used to calculate proportion aware and proportion treated.

### Statistical analysis

All the summary estimates reported reflect weighting to account for the multistage sampling design, thus resulting in nationally-representative estimates. Age-standardization was performed using the age structure of the Thai population registry in 2019 as a standard population. The age-standardized prevalence of hypertension indicators and the demographic and socioeconomic characteristics associated with them were calculated separately using data from each of the four survey cycles from 2004 to 2019–2020. Crude and age-standardized prevalence of hypertension and proportion of awareness, treatment and control among those with hypertension and proportion of control among those treated were calculated and then weighted using survey weights. These proportions were calculated according to sex, age group, area of residence (urban/rural), educational level and wealth index quintiles.

We calculated p-for trend of prevalence across year using linear regression.

Multivariable logistic regression models were used to examine associations of survey variables with hypertension, awareness and control. Covariates tested in the multivariable logistic regression models were selected a priori based on prior literature and included age group, sex, area of residence, education, BMI categories (< 18.5, 18.5-<23, 23-<25, 25-<30, and > = 30 kg/m^2^), physical activity level (low vs. > = moderate level) and wealth index quintiles. Adjusted Odds ratio and 95% confidence interval (CI) were calculated. All the statistical analyses were performed using Stata version16.1 (StataCorp, Texas).

## Results

After applying exclusions, a total of 95,062 participants were included in the study. Proportions by age group, sex, area of residence, education, and wealth index quintiles are described in Tables 1 and 2.

**Table 1:** Characteristics of Thai adults in National Health examination surveys (NHES), 2004 to 2019–2020

	2004	2009	2014	2019–2020
N	37,875	19,309	18,066	19,062
**Sex**				
Men	48.5	48.1	47.7	47.2
Women	51.5	51.9	52.3	52.8
**Age group, year (%)**				
20–39	50	34	33.3	32.5
40–59	34.9	50.1	46.2	44.6
>=60	15.2	15.9	20.5	22.9
**Area of residence**				
Urban	25.5	30.8	45.2	34.1
Rural	74.5	69.2	54.8	65.9
**Education**				
<=primary	69.2	66.8	57.6	50
>=Secondary	30.9	33.2	42.4	50
**Smoking**				
No	65.8	64.3	65	64.6
Ex-smoker	7.8	11.3	14.5	16
Yes	26.5	24.4	20.5	19.5
**Physical activity**				
Moderate to high	77.9	81.8	81.0	69.5
Low	22.1	18.2	19.0	30.5
**Wealth index**				
Q1	21.1	22.5	20.6	21.4
Q2	19.2	17.6	21.9	19.6
Q3	22.5	19.8	19.1	20.5
Q4	17.3	19.8	19.6	18.8
Q5	19.9	20.3	18.8	19.7
**Alcohol consumption in the past 12 months**				
No	46.4	54.5	61	55.1
Yes	53.6	45.5	39	44.9
**BMI (kg/m**^**2**^)				
< 18.5	9.0	6.9	6.7	5.8
18.5-<23	42	38.3	35.3	32.3
23-<25	18.5	18.1	17.9	17.9
25-<30	23.2	27.5	28.7	30.2
>=30	7.3	9.3	11.5	13.7

**Table 2:** Trends and age-standardized and age-category-specific prevalence of hypertension among Thai adults, NHES 2004 to 2019–2020

	2004	2009	2014	2019–2020	*P* for trend
N	37,875	19,309	18,066	19,062	
Age-standardized	27.1(25.0,29.3)	23.1(20.3,25.9)	26.7(24.7,28.7)	25.7(23.5,27.8)	0.96
**Sex**					
Men: Age-standardized	27.9(25.2,30.5)	23.1(19.2,27.0)	27.5(24.9,30.2)	26.8(24.0,29.6)	0.93
Women: Age-standardized	26.5(24.4,28.6)	23.1(21.1,25.1)	25.9(23.6,28.2)	24.6(22.9,26.4)	0.75
**Age group, years (%)**					
20–39 Crude	10.4(8.6,12.2)	7.4(6.3,8.5)	9.8(8.6,11.0)	7.7(6.2,9.3)	0.51
40–59 Crude	31.1(28.3,33.9)	26.1(22.5,29.6)	30.3(27.4,33.2)	25.6(22.6,28.7)	0.44
>=60 Crude	51.6(48.4,54.8)	48.1(42.3,54.0)	53.2(48.5,57.9)	60.6(57.4,63.9)	0.21
**Area of residence**					
Urban: Age-standardized	29.7(27.2,32.2)	27.3(25.3,29.2)	27.5(26.1,28.9)	25.2(23.2,27.1)	0.07
Rural: Age-standardized	26.2(23.8,28.6)	21.1(18.1,24.0)	26.3(23.5,29.0)	26.0(23.3,28.7)	0.77
**Education**					
<=Primary: Age-standardized	27.6(25.3,30.0)	22.6(19.8,25.3)	28.4(26.0,30.9)	27.7(25.1,30.4)	0.70
Secondary: Age-standardized	27.5(25.2,29.8)	25.5(23.0,28.1)	26.2(24.2,28.2)	23.2(21.3,25.0)	0.12
**Wealth index**					
Q1 Age_standardized	na	20.5(17.0,23.9)	26.6(23.7,29.5)	27.3(24.7,29.9)	0.27
Q2 Age_standardized	na	21.2(18.5,24.0)	24.1(20.6,27.5)	25.8(23.3,28.4)	0.09
Q3 Age_standardized	na	22.9(20.1,25.6)	27.0(23.5,30.5)	27.1(24.4,29.9)	0.32
Q4Age_standardized	na	26.0(23.6,28.3)	28.0(25.9,30.1)	24.4(21.4,27.4)	0.70
Q5 Age_standardized	na	26.2(24.0,28.4)	27.4(25.3,29.5)	24.0(21.7,26.4)	0.56

## Primary outcome

### Hypertension prevalence

**Fig. 1:**
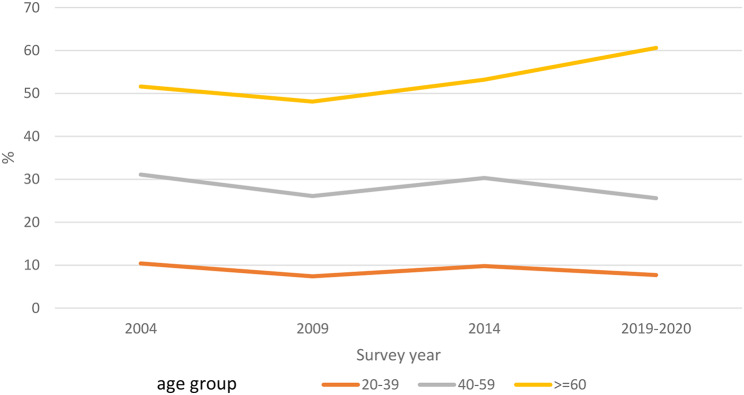
Hypertension prevalence in all Thai adults. Trends in hypertension prevalence from 2004 to 2019–2020 are shown for adults by age groups 20–39, 40–59, and 60 and older. Hypertension prevalence decreased in 2019–2020 for adults age 20–59, but increased for adults age 60 and older

The estimated age-standardized prevalence of hypertension (weighted to represent the Thai population) was 27.1% in 2004, 23.1% in 2009, 26.7% in 2014, and 25.7% in 2019–2020. See Table 2 for age-standardized results and Fig. 1 for age-category-specific prevalence; Supplemental Appendix Table 1 A includes crude and age-standardized results. Among adults with hypertension, the age-standardized estimated mean SBP level was 139.2 mm Hg and the mean DBP level was 90.7 mm Hg in 2004, and 144.2 mm Hg and 84.8 mm Hg, respectively, in 2019–2020 (Supplemental Appendix Table 2A).

### BP control

**Fig. 2:**
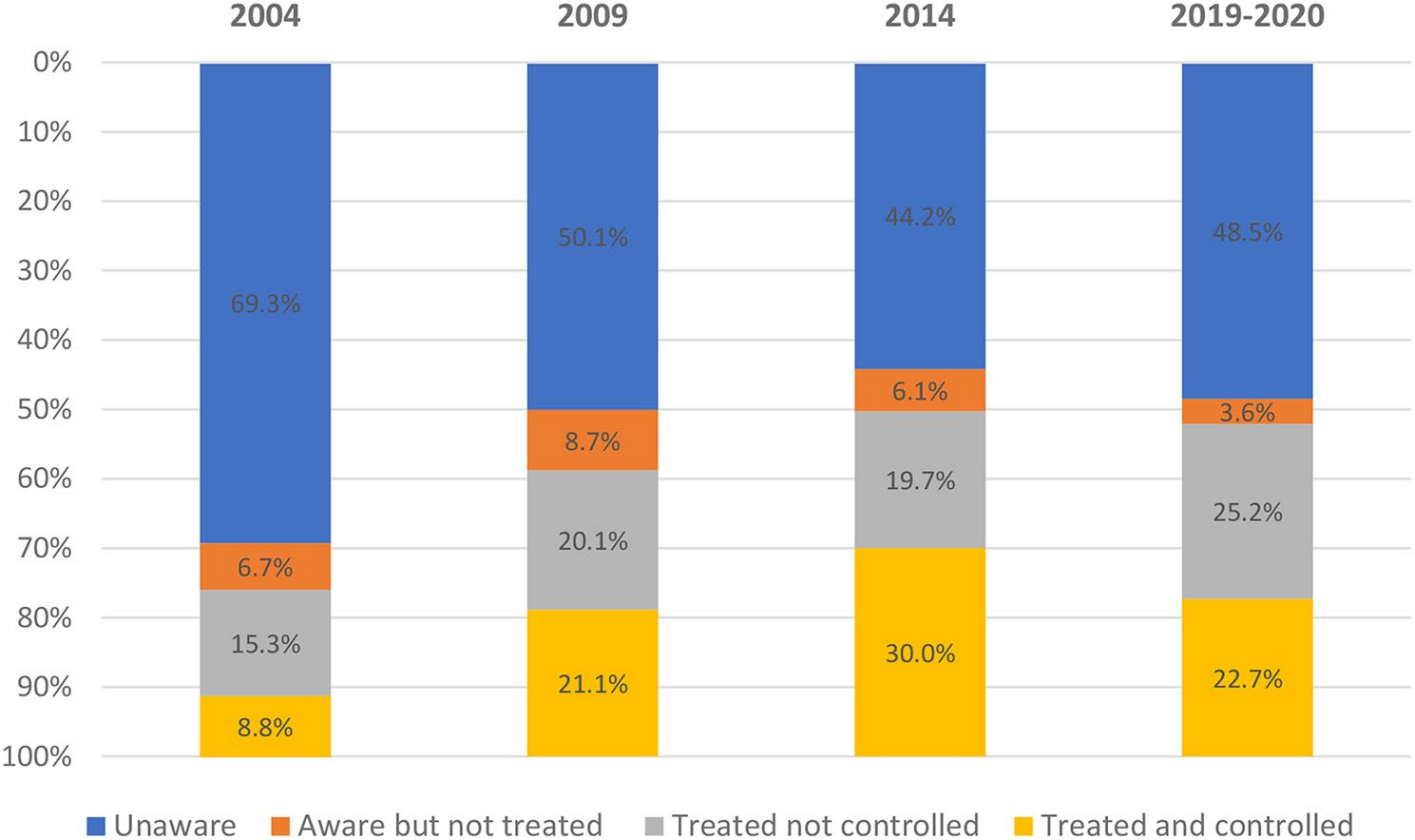
Proportion of Thai adults aged > = 20 years reaching each step of the hypertension care cascade over the years 2004, 2009, 2014 and 2019–2020. Awareness was calculated among adults with hypertension and increased from 2004–2014 then decreased in 2019–2020. Awareness is represented by the sum of the orange, grey and yellow bars. Treatment was calculated among adults with hypertension and increased each survey year. Treatment is represented by the grey and yellow bars. Control was calculated among adults with hypertension and increased from 2004–2014, then decreased in 2019–2020. Control is represented by the yellow bars

Among all adults with hypertension, the proportion with controlled BP increased from 8.8% (95% CI, 7.2-10.3%) in 2004 to 21.0% (95% CI, 18.3-23.8%) in 2009, then increased again to 30.0% (95% CI, 26.3-33.7%) in 2014, and then declined to 22.7% (95% CI,18.6-26.8%) in 2019–2020 (Fig. 2; Table 3A in the Supplemental Appendix).

**Fig. 3:**
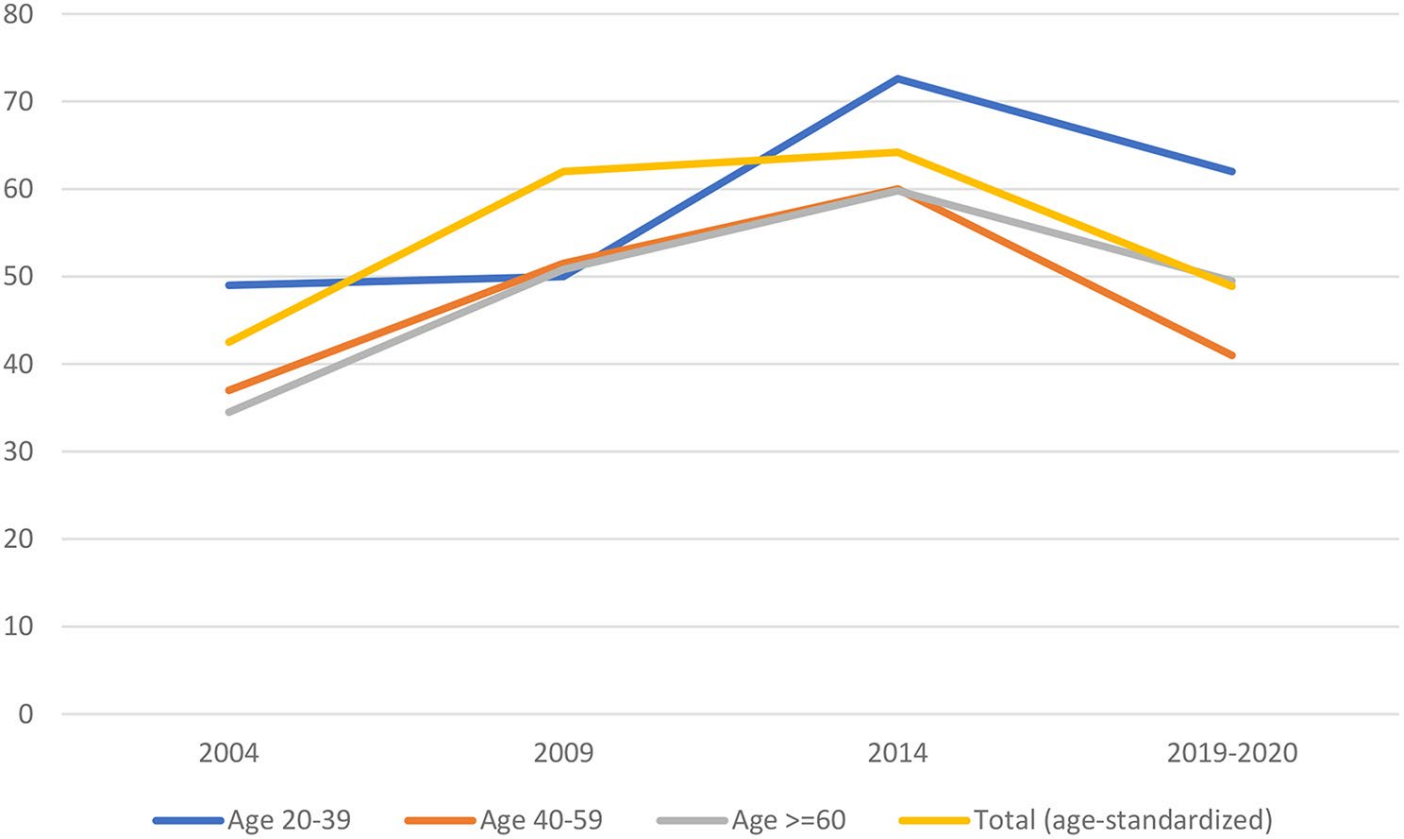
Trends in hypertension control among treated Thai adults, 2004 to 2019–2020. Trends in hypertension control are shown for adults by age groups 20–39, 40–59, and 60 and older, along with the age-standardized total prevalence for all adults. Hypertension control rates increased from 2004–2014 and then decreased in 2019–2020 across all age groups

Among adults taking antihypertensive medication, the estimated proportion with controlled BP increased from 36.5% (95% CI, 32.5-40.5%) in 2004 to 51.1% (95% CI,47.4-54.9%) in 2009, then increased again to 60.4% (95% CI, 56.5%, 64.3% 56.5%, 64.3%) in 2014, and then declined to 47.4% (95% CI, 42.2- 52.5%) in 2019–2020 (Fig. 3; Table 3A in the Supplemental Appendix). The estimated proportions of hypertensive adults with controlled BP by the categories of age group, sex, living area, and education, overall, and restricted to those taking antihypertensive medication appear in Supplemental Appendix Table 3A.

Compared with adults who were aged 20–39 years and 40–59 years in the overall population with hypertension in 2004–2019, controlled BP was more likely among those aged 60 years and older (Adjusted odds ratio, 2.2 [95% CI, 1.31–3.66]). The likelihood of BP control was lower for the lowest wealth index quintiles (Q2, and Q1), compared to the highest (Q5; Adjusted odds ratios 0.6 [95% CI, 0.50-0.84], and 0.7 [95% CI, 0.55-0.92], respectively) (Table 3). Women were more likely than men to have their blood pressure under control (Adjusted odds ratio, 1.6 [95% CI, 1.24–2.06]) (Table 3).

**Table 3:** Adjusted odds ratio (AOR) for factors associated with awareness, treatment and control of blood pressure among Thai adults with hypertension, NHES 2019–2020

Group	Prevalence		Awareness		Treatment		Control	
	AOR	95% CI	AOR	95% CI	AOR	95% CI	AOR	95% CI
**Sex**								
Men	1		1		1		1	
Women	0.7	(0.62,0.79)	1.4	(1.20,1.70)	1.4	(1.20,1.75)	1.6	(1.24,2.06)
**Age groups**								
20–39	1		1		1		1	
40–59	3.6	(2.98,4.38)	2.1	(1.38,3.08)	1.7	(1.13,2.43)	1	(0.63,1.58)
>=60	17	(14.02,21.06)	4.9	(3.31,7.45)	4.5	(3.04,6.64)	2.2	(1.31,3.66)
**Education level**								
<=primary	1.4	(1.20,1.61)	1.1	(0.84,1.33)	1.1	(0.88,1.24)	0.9	(0.76,1.14)
≥Secondary	1		1		1		1	
**Area**								
Urban	1	(0.86,1.25)	1	(0.85,1.33)	1.1	(0.89,1.24)	1.1	(0.81,1.37)
Rural	1		1		1		1	
**Smoking**								
Never	1		1		1		1	
Ever	1.1	(0.80,1.41)	1.3	(1.07,1.51)	1.2	(0.95,1.48)	1.2	(0.93,1.51)
Current	1	(0.77,1.20)	0.9	(0.70,1.12)	0.8	(0.63,0.97)	1	(0.70,1.34)
**Alcohol Drinking (12 m)**								
No	1		1		1		1	
Yes	1.1	(0.94,1.24)	0.7	(0.59,0.85)	0.7	(0.58,0.87)	0.7	(0.64,0.82)
**BMI category (kg/m2)**								
Underweight (< 18.5)	0.70	(0.59, 0.83)	0.82	(0.55, 1.23)	0.81	(0.53,1.23)	0.7	(0.39,1.08)
Normal weight (18.5-<23)	1		1		1		1	
Overweight (23-<25)	1.2	(0.98,1.38)	1.5	(1.17,1.83)	1.3	(1.08,1.61)	1.1	(0.83,1.38)
Obese I (25-<30)	1.44	(1.25,1.64)	1.6	(1.34,2.00)	1.6	(1.28,1.94)	1.1	(0.71,1.40)
ObeseII ( > = 30)	2.5	(2.18,3.0)	2.7	(2.16,3.35)	2.3	(1.85,2.80)	1.5	(1.02,2.23)
**Physical activity**								
Moderate to high	1		1		1		1	
Low	1.04	0.88, 1.23	1.1	0.93, 1.33	1.1	0.93, 1.33	1.14	0.91, 1.44
**Wealth index**								
Q1	1.2	(0.94,1.49)	0.7	(0.54,1.02)	0.7	(0.53,1.03)	0.7	(0.55,0.92)
Q2	1.1	(0.89,1.42)	0.6	(0.52,0.74)	0.6	(0.47,0.80)	0.6	(0.50,0.84)
Q3	1.2	(0.99,1.37)	0.8	(0.64,0.98)	0.8	(0.59,1.15)	0.8	(0.61,1.04)
Q4	1	(0.83,1.18)	1	(0.84,1.29)	1.1	(0.84,1.36)	0.9	(0.68,1.23)
Q5	1		1		1		1	

All model included: sex, age group, area, educational level, smoking, alcohol drink, BMI category, physical activity, and wealth index

### Secondary outcomes

Among adults with hypertension, the proportion who reported that they were aware they had hypertension increased from 30.7% (95% CI, 28.2-33.3%%) in 2004 to 50.0% (95% CI, 45.6-54.2%) in 2009 and then to 55.8% (95% CI, 52.6-59.0%) in 2014, then declined to 51.5% (95% CI, 46.9-56.0%) in 2019–2020 (Fig. 2; Table 4 A in the Supplemental Appendix). In the overall adult population with hypertension in 2004 to 2019–2020, it was estimated that hypertension awareness was more likely in those who were aged 60 years and older and 40–59 compared to their counterparts aged 20–39 (Adjusted odds ratio, 4.9 [95% CI, 3.31–7.45], 2.1 [95% CI, 1.38–3.08], respectively (Table 3). It was estimated that hypertension awareness was more likely in women than men (Adjusted odds ratio, 1.4 [95% CI, 1.20–1.70]).

Among adults with hypertension, the proportion who reported taking antihypertensive medication increased from 24.1% (95% CI, 21.7-26.4%) in 2004 to 41.2% (95% CI, 36.8-45.6%) in 2009 then to 49.7% (95% CI, 46.4-52.9%) in 2014, then to 47.9% (95% CI, 43.7-52.2%) in 2019–2020 (Fig. 2; Table 4 A in the Supplemental Appendix). It was estimated that among adults who reported being aware they had hypertension, the self-reported use of antihypertensive medication was more likely among those aged 60 years and 40–59 compared to adults aged 20–39 (Adjusted odds ratio, 4.5 [95% CI, 3.04–6.64], 1.7 [95% CI, 1.13–2.43], respectively (Table 3). Women were more likely than men to report taking antihypertensive medications (Adjusted odds ratio, 1.4 [95% CI, 1.20–1.75])(Table 3).

## Discussion

Age-standardized prevalence of hypertension has remained relatively unchanged in Thai adults from 2004 to 2019–2020 and remains higher than the global average [9]. Meanwhile, the proportion of Thai adults with controlled BP increased favorably from 2004 until 2014, but decreased by 7% points between 2014 and 2019–2020.

Awareness of hypertension increased by 25% between 2004 and 2014, with the biggest increase occurring between 2004 and 2009. It then dropped by 5% from 2014 to 2019–2020. The sharp increase in awareness from 2004 to 2009 coincides with the launch of a national campaign of community-based blood pressure measurement by trained health volunteers, in addition to a hospital-based opportunistic screening program. Thailand’s universal healthcare coverage (UC) began in 2002 and may have driven early improvements in awareness and control. The UC scheme covers all Thai citizens who are not covered by other health security schemes (e.g. civil servant and social security) and provides treatment services to patients for no charge, which reduces the financial barrier of the patients in access to medical care [10]. 

Despite Thailand’s robust UC and universal screening of high BP for the eligible adult population, half of people with hypertension have not received a diagnosis. This could be due to multiple factors including insufficient opportunistic screening in primary care facilities; lack of clear operational steps to record a confirmed diagnosis of hypertension, leading to high drop out of patients; errors in BP measurement due to non-adherence to BP measurement protocols; lack of adherence to Thai Hypertension Society guidelines for diagnosis, and overreliance on home blood pressure while missing opportunities to improve BP measurement in clinics [11]. 

BP control among adults with hypertension decreased by 7% from 2014 to 2019–2020. A proportion of this decline in control rate was from patients that had started on treatment; 52.6% of adults that were on treatment still had uncontrolled BP in 2019, compared to 39.6% in 2014. This is likely due to non-adherence to the Thai hypertension treatment guidelines. In a 2021 survey of Thai hospitals, almost 30% of patients that required escalation of their medications did not receive a medication adjustment [11]. Furthermore, only 43% of patients that were recommended dual therapy according to the Thai Hypertension guidelines received it. WHO recommends combination therapy as an initial treatment for people with hypertension, preferably a single-pill combination, as it promotes medication adherence and persistence, and BP control [8]. Another contributor to the negative trend in hypertension control may be that patients are seeking care outside of the public sector; an estimated 30–40% of hypertension care is delivered in non-Ministry of Public Health and private sector health care facilities that may not be designed to diagnose and manage chronic conditions like hypertension [4]. Thailand is just one of a group of countries (including the U.S. and Canada) that achieved improved hypertension outcomes, only to fall behind recently [12, 13]. 

With a vigorous response, Thailand can reclaim the past favorable trends in hypertension outcomes. Next steps might include raising public awareness of the importance of hypertension diagnosis and treatment using several channels and targeted messages, awareness raising among health care professionals, and education and electronic decision-support tools to reduce diagnostic and therapeutic inertia. Recent approaches supported by the Thai clinical practice guideline for hypertension include a BP home-monitoring pilot project in selected areas, which has been adopted and supported by the national health Security office. Within the primary care facilities, quality of care can be improved by introducing simple treatment protocols, single-pill combination medicines, and team-based care, as recommended by the 2021 World Health Organization hypertension treatment guidelines [8]. Last, collaboration with the private sector can increase access to standard hypertension care and may improve hypertension outcomes in the working population. Routine collection of hypertension outcomes data using standard indicators will be needed to assess the results of these efforts to drive progress in control of hypertension.

It is important to note that the unfavorable hypertension outcome trends observed in the 2019–2020 national survey do not fully capture the impact of health system challenges from the COVID-19 pandemic that may have compromised hypertension care and affected hypertension outcomes. The COVID-19 pandemic disrupted data collection in the 2019–2020 NHES; the survey started in September 2019 and ran until January 1st, 2020, when the first case of Covid-19 detected in Thailand, at which point 54% of participants were already enrolled. The survey then continued with an additional 30% of participants enrolled until March 19th, 2020 at which point we discontinued the survey because the country went on lockdown. The survey was restarted in June 2020 and enrolled the remaining 16% of participants through October 11th, 2020. However, the Covid-19 pandemic may have hindered access to healthcare and screening services to some extent, leading to reduced awareness and control percentages in the 2019 survey.

This study has several other limitations. First, the participants completed only a single measurement and guidelines recommend obtaining the mean using multiple BP measurements obtained during 2 or more visits [14]. Second, the analysis relied on self-report of hypertension awareness and treatment with antihypertensive medication. Third, the associations found between participant characteristics and hypertension outcomes in logistic regression analyses are cross-sectional in nature and causality can therefore not be inferred from these associations. Fourth, complete information on the duration of hypertension was not available, thereby there was no adjustment regarding this variable. Finally, dietary intake and salt intake were not appropriately available in the data to include in the model, which might have contributed to some degree of residual confounding.

In summary, at the outset of the twenty-first century, Thailand demonstrated impressive gains in hypertension outcomes, with increased awareness, treatment, and control. The country experienced decay in these favorable trends for awareness and control in the last, 2019–2020, NHES survey. Thailand will benefit from a concerted effort to return to the favorable, improving trends of the decade 2004 to 2014. Effective treatment and control of hypertension in Thai adults will help save lives by preventing heart attack and stroke. Thailand has set a target to reduce premature mortality from CVD and other noncommunicable diseases (NCDs) by 25% by 2025.

## Conclusions

Despite universal coverage, the prevalence of hypertension continues to remain high in Thailand with nearly half of adults with hypertension still unaware of their diagnosis. Hypertension control has decreased by 7% points since the last NHES assessment partly due to a decline in hypertension control among treated patients (from 60.4% in 2014 to 47.4% in 2019–2020). Urgent and rigorous measures are needed to reduce diagnosis and treatment inertia in Thailand.

## Electronic supplementary material

Below is the link to the electronic supplementary material.

Supplementary Material 1

## Data Availability

Supplemental tables can be found in the supplemental appendix. Additional data are available from the corresponding author on reasonable request.

## Abbreviations

BP: Blood pressure
CVD: Cardiovascular disease
DBP: Diastolic blood pressure
IHD: Ischemic heart disease
NCD: Non-communicable disease
NHES: National Health Examination Survey
SBP: Systolic blood pressure
UC: Universal coverage
