# Supplementary material for: Trends in hypertension prevalence, awareness, treatment, and control in the Thai population, 2004 to 2020
Source: BMC Public Health. 2024 Nov 13;24:3149. doi: 10.1186/s12889-024-20643-1 (PMC11562084; doi:10.1186/s12889-024-20643-1)
Supplement: Supplementary file 1 — Supplementary Material 1 [file 12889_2024_20643_MOESM1_ESM.docx]

**SUPPLEMENTAL APPENDIX**

**Title**: Trends in hypertension prevalence, awareness, treatment, and control in the Thai population, 2004 to 2019-2020

**Authors:**

Wichai AEKPLAKORN^a*^, MD, PhD, Suwat CHARIYALERTSAK^b^, MD, Pattapong KESSOMBOON^c^, MD, Sawitri ASSANANGKORNCHAI^d^, MD, Surasak TANEEPANICHSKUL^e^, MD, Allison GOLDSTEIN^f^, MPH, Danielle CAZABON^f^, MPH Nareemarn NEELAPAICHIT^g^, DrPH

^a^Department of Community Medicine, Faculty of Medicine Ramathibodi Hospital, Mahidol University, Rama VI Rd., Ratchathewi, Bangkok, Thailand

University, Bangkok, Thailand

^b^Faculty of Public Health, Chiang Mai University, Chiang Mai, Thailand

^c^Faculty of Medicine, Khon Kaen University, Khon Kaen, Thailand

^d^Epidemiology Unit, Faculty of Medicine, Prince of Songkla University, Songkhla, Thailand

^e^College of Public Health Sciences, Chulalongkorn University, Bangkok, Thailand

^f^Resolve To Save Lives

^g^Ramathibodi School of Nursing, Faculty of Medicine, Ramathibodi Hospital, Mahidol

**SUPPLEMENTAL APPENDIX TABLE OF CONTENTS**

**Supplemental Appendix Tables (Pages 3-9)**

- Supplemental Appendix Table 1A. Trends, crude and age-standardized (95% CI) prevalence of hypertension among Thai adults, NHES 2004 to 2019-2020.
- Supplemental Appendix Table 2A. Crude and Age-standardized mean systolic blood pressure (SBP) and diastolic blood pressure (DBP) and BP categories among Thai adults, NHES 2004 to 2019-2020.
- Supplemental Appendix Table 3A. trends, age-standardized proportion of control among Thai adults with hypertension, NHES 2004 to 2019-2020.
- Supplemental Appendix Table 4A. trends, age-standardized proportion of awareness, treatment among Thai adults with hypertension, NHES 2004 to 2019-2020.

**SUPPLEMENTAL APPENDIX TABLES**

**Supplemental Appendix Table 1A. Trends, crude and age-standardized (95% CI) prevalence of hypertension among Thai adults, NHES 2004 to 2019-2020.**

|  | 2004 | 2009 | 2014 | 2019-2020 |
| --- | --- | --- | --- | --- |
| N | 37875 | 19309 | 18066 | 19812 |
| Total |  |  |  |  |
| crude | 23.9(21.7,26.0) | 23.2(19.8,26.6) | 28.2(25.8,30.5) | 27.8(25.3,30.3) |
| Age-standardized | 27.1(25.0,29.3) | 23.1(20.3,25.9) | 26.7(24.7,28.7) | 25.7(23.5,27.8) |
| **Sex** |  |  |  |  |
| Men: Crude | 25.2(22.5,27.8) | 23.5(18.7,28.3) | 29.4(26.6,32.1) | 29.5(26.3,32.7) |
| Age-standardized | 27.9(25.2,30.5) | 23.1(19.2,27.0) | 27.5(24.9,30.2) | 26.8(24.0,29.6) |
| Women :Crude | 22.7(20.7,24.6) | 22.9(20.6,25.3) | 27.1(24.2,29.9) | 26.4(24.2,28.6) |
| Age-standardized | 26.5(24.4,28.6) | 23.1(21.1,25.1) | 25.9(23.6,28.2) | 24.6(22.9,26.4) |
| **Age group, year (%)** |  |  |  |  |
| 20-39 Crude | 10.4(8.6,12.2) | 7.4(6.3,8.5) | 9.8(8.6,11.0) | 7.7(6.2,9.3) |
| 40-59 Crude | 31.1(28.3,33.9) | 26.1(22.5,29.6) | 30.3(27.4,33.2) | 25.6(22.6,28.7) |
| >=60 Crude | 51.6(48.4,54.8) | 48.1(42.3,54.0) | 53.2(48.5,57.9) | 60.6(57.4,63.9) |
| **Area of residence** |  |  |  |  |
| Urban:Crude | 26.6(24.0,29.2) | 29.0(25.9,32.1) | 28.1(25.7,30.5) | 28.1(25.4,30.8) |
| Age-standardized | 29.7(27.2,32.2) | 27.3(25.3,29.2) | 27.5(26.1,28.9) | 25.2(23.2,27.1) |
| Rural: Crude | 22.9(20.6,25.3) | 20.6(17.4,23.9) | 28.2(25.2,31.3) | 27.7(24.6,30.8) |
| Age-standardized | 26.2(23.8,28.6) | 21.1(18.1,24.0) | 26.3(23.5,29.0) | 26.0(23.3,28.7) |
| **Education** |  |  |  |  |
| <=primary:Crude | 27.9(25.5,30.4) | 26.9(22.7,31.1) | 35.8(32.8,38.8) | 40.1(36.6,43.6) |
| Age-standardized | 27.6(25.3,30.0) | 22.6(19.8,25.3) | 28.4(26.0,30.9) | 27.7(25.1,30.4) |
| Secondary:Crude | 14.7(12.9,16.5) | 15.7(12.5,19.0) | 17.9(15.7,20.2) | 15.5(13.3,17.7) |
| Age-standardized | 27.5(25.2,29.8) | 25.5(23.0,28.1) | 26.2(24.2,28.2) | 23.2(21.3,25.0) |
| **Wealth index** |  |  |  |  |
| Q1 crude | na | 21.7(17.2,26.3) | 30.4(26.9,33.8) | 28.2(25.4,31.1) |
| Q1 Age-standardized | na | 20.5(17.0,23.9) | 26.6(23.7,29.5) | 27.3(24.7,29.9) |
| Q2crude | na | 20.6(17.2,23.9) | 26.0(22.2,29.9) | 28.9(25.7,32.0) |
| Q2 Age-standardized | na | 21.2(18.5,24.0) | 24.1(20.6,27.5) | 25.8(23.3,28.4) |
| Q3crude | na | 21.8(18.6,24.9) | 27.8(24.1,31.6) | 29.8(26.2,33.3) |
| Q3 Age-standardized | na | 22.9(20.1,25.6) | 27.0(23.5,30.5) | 27.1(24.4,29.9) |
| Q4 crude | na | 26.0(22.8,29.2) | 29.9(27.3,32.5) | 26.3(22.4,30.1) |
| Q4Age-standardized | na | 26.0(23.6,28.3) | 28.0(25.9,30.1) | 24.4(21.4,27.4) |
| Q5crude | na | 26.6(23.5,29.6) | 27.2(24.6,29.8) | 26.1(23.3,28.9) |
| Q5 Age-standardized | na | 26.2(24.0,28.4) | 27.4(25.3,29.5) | 24.0(21.7,26.4) |

**Supplemental Appendix Table 2A. Crude and Age-standardized mean systolic blood pressure (SBP) and diastolic blood pressure (DBP) and BP categories among Thai adults, NHES 2004 to 2019-2020.**

|  | 2004 | 2009 | 2014 | 2019-2020 |
| --- | --- | --- | --- | --- |
| Total population | 37875 | 19309 | 18066 | 19062 |
| N |  |  |  |  |
| Mean level (95%CI), mmHg | Mean (95%CI) | Mean (95%CI) | Mean (95%CI) | Mean (95%CI) |
| SBP: Crude | 119.4(118.3,120.6) | 122.9(121.6,124.3) | 123.6(121.8,125.4) | 124.9(123.5,126.3) |
| Age-standardized | 121.0(119.8,122.2) | 122.8(121.7,124.0) | 122.9(121.3,124.4) | 123.6(122.3,125.0) |
| DBP:Crude | 76.9(76.1,77.8) | 76.0(75.2,76.8) | 76.5(75.8,77.1) | 73.2(72.6,73.7) |
| Age-standardized | 77.4(76.6,78.3) | 75.3(74.6,76.0) | 75.8(75.2,76.4) | 72.5(71.9,73.1) |
| SBP/DBP category, % (95%CI), mmHg |  |  |  |  |
| <120/<80: Crude | 2.6(2.0,3.3) | 4.9(3.9,6.0) | 7.2(5.6,9.2) | 4.7(3.6,6.2) |
| Age-standardized | 2.9(2.0,4.1) | 5.1(3.2,7.9) | 4.9(3.6,6.6) | 5.0(3.3,7.6) |
| 120-129/<80: Crude | 1.2(0.9,1.5) | 5.1(3.9,6.6) | 7.6(6.5,8.9) | 5.9(4.5,7.7) |
| Age-standardized | 0.9(0.7,1.1) | 3.5(2.7,4.4) | 6.1(5.0,7.3) | 3.6(2.7,4.7) |
| 130-139/80-89: Crude | 5.1(4.2,6.0) | 11.5(9.8,13.4) | 15.2(13.4,17.1) | 12.1(10.1,14.3) |
| Age-standardized | 4.0(3.3,4.7) | 8.3(7.2,9.6) | 11.8(10.2,13.7) | 10.1(7.6,13.2) |
| 140-159/90-99: Crude | 63.2(60.9,65.5) | 55.6(53.0,58.2) | 49.4(47.1,51.8) | 54.7(51.1,58.4) |
| Age-standardized | 67.6(64.6,70.5) | 64.5(61.0,67.8) | 57.7(52.6,62.5) | 61.2(55.0,67.1) |
| >=160/100-179/100: Crude | 25.1(23.0,27.3) | 20.8(18.6,23.2) | 18.5(16.2,20.9) | 21.1(18.6,23.8) |
| Age-standardized | 22.4(20.2,24.9) | 17.1(15.1,19.2) | 18.0(14.3,22.4) | 19.0(15.1,23.5) |
| >=180/100: Crude | 2.9(2.4,3.5) | 2.2(1.5,3.1) | 2.1(1.6,2.8) | 1.5(1.0,2.0) |
| Age-standardized | 2.2(1.8,2.7) | 1.6(1.1,2.3) | 1.6(1.1,2.3) | 1.2(0.8,1.7) |
| Adults with hypertension |  |  |  |  |
| N | 14940 | 6708 | 6747 | 7136 |
| Mean level (95%CI), mmHg | Mean (95%CI) | Mean (95%CI) | Mean (95%CI) | Mean (95%CI) |
| SBP: Crude | 141.6(140.5,142.7) | 145.1(143.9,146.3) | 142.8(140.9,144.8) | 146.9(145.6,148.3) |
| Age-standardized | 139.2(138.0,140.5) | 142.7(141.6,143.9) | 142.4(140.7,144.1) | 144.2(143.2,145.2) |
| DBP:Crude | 90.5(89.8,91.2) | 87.2(86.7,87.6) | 85.9(85.2,86.7) | 80.6(80.0,81.1) |
| Age-standardized | 90.7(90.1,91.2) | 88.5(87.7,89.2) | 88.3(87.5,89.0) | 84.8(84.2,85.4) |
| SBP/DBP category,% (95%CI), mmHg |  |  |  |  |
| <120/<80: Crude | 2.6(2.0,3.3) | 4.9(3.9,6.0) | 7.2(5.6,9.2) | 4.7(3.6,6.2) |
| Age-standardized | 2.9(2.0,4.1) | 5.1(3.2,7.9) | 4.9(3.6,6.6) | 5.0(3.3,7.6) |
| 120-129/<80: Crude | 1.2(0.9,1.5) | 5.1(3.9,6.6) | 7.6(6.5,8.9) | 5.9(4.5,7.7) |
| Age-standardized | 0.9(0.7,1.1) | 3.5(2.7,4.4) | 6.1(5.0,7.3) | 3.6(2.7,4.7) |
| 130-139/80-89: Crude | 5.1(4.2,6.0) | 11.5(9.8,13.4) | 15.2(13.4,17.1) | 12.1(10.1,14.3) |
| Age-standardized | 4.0(3.3,4.7) | 8.3(7.2,9.6) | 11.8(10.2,13.7) | 10.1(7.6,13.2) |
| 140-159/90-99: Crude | 63.2(60.9,65.5) | 55.6(53.0,58.2) | 49.4(47.1,51.8) | 54.7(51.1,58.4) |
| Age-standardized | 67.6(64.6,70.5) | 64.5(61.0,67.8) | 57.7(52.6,62.5) | 61.2(55.0,67.1) |
| >=160/100-179/100: Crude | 25.1(23.0,27.3) | 20.8(18.6,23.2) | 18.5(16.2,20.9) | 21.1(18.6,23.8) |
| Age-standardized | 22.4(20.2,24.9) | 17.1(15.1,19.2) | 18.0(14.3,22.4) | 19.0(15.1,23.5) |
| >=180/100: Crude | 2.9(2.4,3.5) | 2.2(1.5,3.1) | 2.1(1.6,2.8) | 1.5(1.0,2.0) |
| Age-standardized | 2.2(1.8,2.7) | 1.6(1.1,2.3) | 1.6(1.1,2.3) | 1.2(0.8,1.7) |

**Supplemental Appendix Table 3A. Trends, crude and age-standardized proportion of control among Thai adults with hypertension, NHES 2004 to 2019-2020.**

|  | 2004 | 2009 | 2014 | 2019-2020 |
| --- | --- | --- | --- | --- |
| N |  |  |  |  |
| **Control among all with hypertension** | % (95% CI) | % (95% CI) | % (95% CI) | % (95% CI) |
| **Total** |  |  |  |  |
| Crude | 8.8(7.2,10.3) | 21.0(18.3,23.8) | 30.0(26.3,33.7) | 22.7(18.6,26.8) |
| Age-standardized | 7.7(6.2,9.2) | 16.6(13.8(19.4) | 22.7(19.5,26.0) | 18.7(14.9,22.4) |
| Sex |  |  |  |  |
| Men:Crude | 5.9(4.5,7.2) | 14.5(11.8,17.1) | 22.6(18.6,26.7) | 17.1(13.4,20.7) |
| Age-standardized | 4.8(3.7,5.9) | 10.1(8.8,11.5) | 16.6(12.9,20.3) | 12.3(9.4,15.2) |
| Women:Crude | 11.8(9.9,13.7) | 27.3(23.4,31.3) | 37.2(33.3,41.2) | 28.4(23.8,32.9) |
| Age-standardized | 10.4(8.1,12.7) | 22.7(17.8,27.6) | 28.5(24.9,32.1) | 24.7(19.7,29.6) |
| Age group, year (%) |  |  |  |  |
| 20-39:Crude | 3.0(1.4,4.5) | 7.3(3.7,10.9) | 12.4(8.3,16.5) | 14.6(8.5,20.7) |
| 40-59:Crude | 8.9(6.7,11.0) | 19.5(16.3,22.8) | 27.3(23.8,30.7) | 15.0(11.2,18.7) |
| >=60:Crude | 12.5(11.0,14.1) | 28.2(25.0,31.4) | 38.8(32.7,44.8) | 30.6(26.0,35.1) |
| Area of residence |  |  |  |  |
| Urban:Crude | 10.8(8.0,13.6) | 24.2(20.5,27.9) | 31.9(28.8,35.0) | 24.5(18.6,30.4) |
| Age-standardized | 9.1(6.9,11.3) | 18.1(15.4,20.9) | 25.6(20.9,30.4) | 20.4(16.1,24.7) |
| Rural:Crude | 8.0(6.5,9.4) | 19.1(16.6,21.6) | 28.4(23.5,33.3) | 21.8(17.6,25.9) |
| Age-standardized | 7.1(5.4,8.8) | 15.4(12.2,18.7) | 20.9(17.6,24.1) | 17.8(13.7,21.8) |
| Education |  |  |  |  |
| <primary:Crude | 9.4(7.9,11.0) | 22.5(19.4,25.5) | 32.0(27.6,36.3) | 24.1(20.0,28.3) |
| Age-standardized | 8.1(5.8,10.5) | 14.9(12.7,17.0) | 23.9(18.9,29.0) | 16.2(11.7,20.7) |
| >=Secondary:Crude | 5.9(3.6,8.3) | 16.7(13.5,19.9) | 24.7(19.8,29.7) | 19.0(13.7,24.4) |
| Age-standardized | 7.6(5.4,9.7) | 17.5(14.2,20.8) | 25.3(22.0,28.5) | 20.9(16.8,24.9) |
| **Control among treated** | % (95% CI) | % (95% CI) | % (95% CI) | % (95% CI) |
| **Total** |  |  |  |  |
| Crude | 36.5(32.5,40.5) | 51.1(47.4,54.9) | 60.4(56.5,64.3) | 47.4(42.2,52.5) |
| Age-standardized | 42.5(34.2,50.9) | 62.0(58.0,66.1) | 64.2(55.9,72.4) | 48.9(39.8,57.9) |
| Sex |  |  |  |  |
| Men:Crude | 33.7(28.4,39.0) | 47.5(43.1,51.8) | 56.7(51.8,61.6) | 43.3(37.5,49.0) |
| Age-std | 36.3(22.7,49.9) | 60.1(55.3,64.8) | 59.9(43.8,76.0) | 40.9(31.0,50.9) |
| Women:Crude | 38.1(34.1,42.0) | 53.2(48.6,57.8) | 62.8(58.5,67.1) | 50.2(44.8,55.7) |
| Age-standardized | 48.3(40.3,56.4) | 63.9(57.9,69.8) | 68.2(61.6,74.7) | 56.3(46.7,65.8) |
| Age group, year (%) |  |  |  |  |
| 20-39:Crude | 49.0(32.1,65.9) | 50.0(19.7,80.3) | 72.6(63.1,82.1) | 62.0(48.7,75.4) |
| 40-59:crude | 37.0(30.9,43.1) | 51.5(45.6,57.5) | 60.0(55.8,64.2) | 41.0(33.7,48.2) |
| >=60:Crude | 34.5(31.6,37.5) | 50.8(47.0,54.6) | 59.8(54.5,65.1) | 49.5(44.8,54.1) |
| Area of residence |  |  |  |  |
| Urban:Crude | 36.9(31.2,42.7) | 51.8(48.4,55.2) | 62.3(58.2,66.4) | 48.2(40.6,55.8) |
| Age-standardized | 37.7(29.4,46.1) | 59.7(55.3,64.1) | 73.3(69.5,77.0) | 53.2(40.9,65.5) |
| Rural:Crude | 36.2(31.7,40.8) | 50.6(45.4,55.8) | 58.7(53.4,64.0) | 46.9(41.9,51.9) |
| Age-standardized | 44.5(33.2,55.8) | 55.3(48.6,62.0) | 57.3(47.4,67.3) | 47.2(37.7,56.7) |
| Education |  |  |  |  |
| <primary:Crude | 36.8(32.9,40.8) | 51.9(47.8,56.0) | 59.2(55.1,63.4) | 47.1(41.9,52.3) |
| Age-standardized | 43.0(33.3,52.8) | 49.1(41.8,56.4) | 62.7(57.6,67.8) | 41.1(33.1,49.2) |
| >=Secondary:Crude | 34.5(27.1,41.9) | 48.2(42.6,53.8) | 64.9(59.4,70.4) | 48.3(40.4,56.2) |
| Age-standardized | 40.8(33.9,47.8) | 62.2(57.6,66.8) | 67.5(57.0,77.9) | 52.2(41.4,62.9) |

**Supplemental Appendix Table 4A. Trends, crude and age-standardized proportion of awareness, treatment among Thai adults with hypertension, NHES 2004 to 2019-2020.**

|  | 2004 | 2009 | 2014 | 2019-2020 |
| --- | --- | --- | --- | --- |
| N | 14940 | 6708 | 6747 | 7136 |
| **Awareness** | % (95% CI) | % (95% CI) | % (95% CI) | % (95% CI) |
| **Total** |  |  |  |  |
| Crude | 30.7(28.2,33.3) | 50.0(45.6,54.2) | 55.8(52.6,59.0) | 51.5(46.9,56.0) |
| Age-standardized | 25.7(23.5,27.8) | 41.2(36.2,46.1) | 43.5(39.3,47.6) | 40.2(34.8,45.5) |
| Sex |  |  |  |  |
| Men:Crude | 23.7(20.9,26.5) | 39.8(33.2,46.3) | 46.5(42.7,50.3) | 43.5(38.2,48.8) |
| Age-standardized | 20.2(17.8,22.5) | 29.9(25.5,34.4) | 35.1(30.9,39.3) | 32.3(27.3,37.3) |
| Women:Crude | 38.1(35.3,40.9) | 59.5(55.8,63.2) | 64.9(61.5,68.4) | 59.5(55.6,63.3) |
| Age-standardized | 30.8(27.8,33.8) | 51.7(43.8,59.6) | 51.3(45.8,56.8) | 47.5(41.0,54.1) |
| Age group, year (%) |  |  |  |  |
| 20-39:Crude | 9.9(7.1,12.7) | 24.3(19.2,29.4) | 25.4(17.0,33.8) | 24.8(16.2,33.3) |
| 40-59:Crude | 31.7(28.7,34.7) | 47.2(42.2,52.3) | 51.5(48.2,54.8) | 41.9(36.4,47.4) |
| >=60:Crude | 43.3(41.3,45.4) | 62.8(59.1,66.5) | 70.3(66.1,74.6) | 64.2(60.1,68.4) |
| Area of residence |  |  |  |  |
| Urban:Crude | 35.7(31.9,39.5) | 55.6(49.1,62.2) | 58.3(55.6,60.9) | 54.3(48.0,60.6) |
| Age-standardized | 29.1(25.8,32.3) | 41.9(36.7,47.2) | 48.2(42.0,54.4) | 42.1(37.7,46.5) |
| Rural:Crude | 28.8(26.4,31.2) | 46.3(43.9,48.7) | 53.7(49.8,57.7) | 50.0(45.3,54.7) |
| Age-standardized | 24.2(22.0,26.4) | 39.4(33.7,45.2) | 40.8(36.4,45.1) | 39.0(32.5,45.5) |
| Education |  |  |  |  |
| <primary:Crude | 32.2(29.7,34.8) | 51.6(47.5,55.8) | 59.4(55.8,62.9) | 54.7(50.4,58.9) |
| Age-standardized | 25.5(22.3,28.7) | 36.5(33.5,39.5) | 42.6(37.3,48.0) | 41.2(33.3,49.1) |
| >=Secondary:Crude | 24.4(20.3,28.4) | 43.9(37.1,50.7) | 46.0(40.2,51.9) | 43.3(35.8,50.8) |
| Age-standardized | 28.7(25.5,31.8) | 43.7(37.0,50.3) | 45.5(40.8,50.2) | 41.7(35.3,48.0) |
| **Treatment** | % (95% CI) | % (95% CI) | % (95% CI) | % (95% CI) |
| **Total** |  |  |  |  |
| Crude | 24.1(21.7,26.4) | 41.2(36.8,45.6) | 49.7(46.4,52.9) | 47.9(43.7,52.2) |
| Age-standardized | 19.8(17.8,21.8) | 31.1(27.3,34.8) | 36.9(33.7,40.1) | 37.4(32.6,42.3) |
| Sex |  |  |  |  |
| Men:Crude | 17.4(15.1,19.7) | 30.5(24.6,36.5) | 39.9(35.7,44.2) | 39.4(35.0,43.8) |
| Age-standardized | 14.1(12.4,15.8) | 21.5(17.9,25.0) | 29.0(25.2,32.9) | 29.4(25.1,33.7) |
| Women:Crude | 31.0(28.2,33.8) | 51.4(47.0,55.8) | 59.3(56.4,62.2) | 56.4(52.4,60.5) |
| Age-standardized | 25.1(22.1,28.1) | 40.1(35.1,45.1) | 44.2(40.6,47.9) | 44.9(38.8,51.1) |
| Age group, year (%) |  |  |  |  |
| 20-39:Crude | 6.0(4.0,8.1) | 14.6(9.0,20.2) | 17.0(12.3,21.8) | 23.6(15.9,31.2) |
| 40-59:Crude | 23.9(21.2,26.6) | 37.9(32.8,43.0) | 45.5(42.1,48.8) | 36.5(32.1,40.9) |
| >=60:Crude | 36.3(34.0,38.5) | 55.5(51.8,59.3) | 64.8(59.9,69.8) | 61.7(57.6,65.9) |
| Area of residence |  |  |  |  |
| Urban:Crude | 29.2(25.7,32.7) | 46.7(40.3,53.2) | 51.2(48.9,53.6) | 50.8(45.3,56.3) |
| Age-standardized | 23.5(20.9,26.1) | 33.6(29.0,38.1) | 38.7(34.2,43.1) | 39.1(35.6,42.7) |
| Rural:Crude | 22.0(19.8,24.2) | 37.7(34.4,41.0) | 48.4(44.1,52.7) | 46.4(41.8,51.1) |
| Age-standardized | 18.3(16.1,20.4) | 29.1(25.2,33.0) | 35.9(32.4,39.3) | 36.3(30.4,42.3) |
| Education |  |  |  |  |
| <primary:Crude | 25.6(23.3,27.9) | 43.3(39.0,47.6) | 53.9(50.1,57.8) | 51.2(46.9,55.5) |
| Age-standardized | 20.2(17.3,23.1) | 29.1(26.4,31.8) | 38.4(32.9,44.0) | 38.5(30.8,46.2) |
| >=Secondary:Crude | 17.2(13.6,20.8) | 34.7(27.4,41.9) | 38.1(33.1,43.1) | 39.4(33.4,45.4) |
| Age-standardized | 21.7(19.1,24.3) | 34.3(29.8,38.7) | 37.3(34.4,40.2) | 38.8(33.5,44.1) |
